# Distribution of self-reported health in India: The role of income and geography

**DOI:** 10.1371/journal.pone.0279999

**Published:** 2023-01-27

**Authors:** Ila Patnaik, Renuka Sane, Ajay Shah, S. V. Subramanian

**Affiliations:** 1 National Institute of Public Finance and Policy, Delhi, India; 2 xKDR Forum, Mumbai, Maharashtra, India; 3 Harvard University, Cambridge, MA, United States of America; Indian Institute of Technology Kanpur, INDIA

## Abstract

An important new large-scale survey database is brought to bear on measuring and analysing self-reported health in India. The most important correlates are age, income and location. There is substantial variation of health across the 102 ‘homogeneous regions’ within the country, after controlling for household and individual characteristics. Higher income is correlated with better health in only 40% of India. We create novel maps showing regions with poor health, that is attributable to the location, that diverge from the conventional wisdom. These results suggest the need for epidemiological studies in the hotspots of ill-health and in regions where higher income does not correlate with improved health.

## Introduction

Health policy is ultimately about creating conditions in which people are healthy. The wellness of the people is the outcome of interest. Many plausible health outcome measures reflect different dimensions of wellness. In the Indian health literature, infant and maternal mortality are the dominant measures which have been employed. For example, [1] find that improved prenatal care impacts neonatal and infant mortality, while [2] find that despite noticeable improvements in maternal health care utilisation since the implementation of National Rural Health Mission (NRHM) in 2005, India did not achieve the target of millennium development goal to reduced maternal mortality ratio by 2015. However, maternal and child mortality represent a subset of the population, and narrow government programs have influenced these metrics without reshaping the broader health of the population.

Medical researchers are able to use objective metrics from medical tests, such as blood pressure, hypertension, anemia, diabetes etc. as a measure of the health of an individual [3–5]. Aggregate measures of disease burden also includes measurement of Disability Adjusted Life Years (DALYs) which captures the reduction in life expectancy and the diminished quality of life [6]. However, such measurement, done consistently across time and space, involves the construction of complex datasets which is infeasible in less developed countries.

Death can be accurately measured, which suggests pathways for health outcomes measurement through death rates and longevity. For example, [7] calculate a M-cure for India, while [8] study the mortality from cervical cancer in Indian states, while [9] use survey data to study links between household economic status and adult mortality. Measures based on mortality in the overall population are impeded by the limitations of official mortality-related statistics. [10] provides a description of the limitations of official statistics.

One path to measuring the health status of an individual is to to directly ask individuals to assess their own health. This is termed Self-Report Health (SRH). For example, the WHO has used the question: *In general, how would you rate your health today?*. Responses to such a survey question can be binary, which naturally suggests an *ill-health rate* which is the fraction of a given set of people who report that their self reported health is not good. Alternatively, a five-point scale can be used, e.g. *In general, would you say that you health is excellent, very good, good, fair, or poor?*.

When compared with objective measures based on medical tests, SRH measurement is inexpensive and less intrusive. From the viewpoint of the foundations of human welfare, asking a person if they are feeling well is of essence [11]. At the same time, SRH suffers from four limitations:

1. SRH inevitably introduces a psychological filter in determining whether there is ill health. For example, [12] find that nearly 40% of respondents change their answers between two interviews done just one month apart, consistency in answers was not influenced by health events, and reliability was worse for disadvantaged groups. There also appears to be a gender gap in reporting of health [13–15]. The under-reporting seems to hold even after controlling for objective health measures [16]. These psychological factors might be correlated with wealth: the tooth ache or fever that makes an upper class person report ill-health might not trigger the same response from a poor person.
2. The precise phrasing of the SRH question in a household survey matters in interpreting the results. Many different designs of the question can be applied, e.g. *Are you feeling well today?* or *Have you been healthy in the last month?* or *In the last week, were you unwell for at least one day?*. As a consequence, the numerical values of the SRH rate are not comparable across survey datasets.
3. The SRH measure also suffers from limitations on comparability between age-groups. For example infants partly cannot communicate their health status to the person responding the survey, and forms of behaviour can be misinterpreted. Further, these other persons (e.g. parents) can be more anxious when their children are very young in contrast to only young.
4. The SRH approach should not be used for specific morbidities. Disorders like diabetes are not discerned by the person for many years. For example, [17] find that the self reported incidence of hypertension and lung disease underestimate the disease burden in India. Other studies find that the prevalence of non communicable diseases is under-reported when viewed through SRH, especially among the poor [18, 19].
5. Access to sound health care, and particularly testing, is likely to influence perceived health. This could generate more accurate answers in households with more access to health care.

While SRH has flaws, it contains useful information about the health of an individual. A successful empirical literature has utilised SRH in health research. It correlates with objective health outcome measures [20, 21]. It does reasonably well on predicting mortality, especially among elderly populations in developed countries [22–25]. In India, [21] use the 2002 WHS data and find that SRH is a useful measure. Similarly [26] use the NFHS as well as the NSS data and find that the use of self-rated ill health has validity in relationship to socioeconomic status. [20] finds a correlation between SRH and disease burden in China.

As a consequence, over the years, SRH measurement has emerged in many survey datasets, such as *World Health Survey 2002 and SAGE 2007* conducted by WHO, the *Health and Retirement study (HRS)* and the *National Health and Nutrition Examination Survey (NHANES)* in the US, the *Survey of Health, Ageing and Retirement in Europe (SHARE)*, the *English Longitudinal Study of Ageing (ELSA)* in the UK. The HRS largely samples the elderly, while the NHANES samples adults and children, and they have used a five-point scale with the categories excellent, very good, good, fair and poor. The ELSA uses a slightly different five point measure: very good, good, fair, bad and very bad. Another example is the SF-36, a short-form 36-item questionnaire developed by the Rand Corporation for medical outcomes measurement in in the US. It is a patient-reported survey of health as measured along eight multi-item categories. Each of these datasets has resulted in evidence about SRH which has fed into a substantial downstream literature. For example, see the literature on understanding the link between income inequality and self-reported health [27–30].

In this paper, we exploit the Consumer Pyramids Household Survey (CPHS), a longitudinal dataset which measures about 170,000 households, three times a year. There is one other panel data set in India, the Indian Human Development Survey (IHDS), where households are observed 10 years apart. While a great deal of the health literature in India is based on the National Sample Survey Organisation (NSSO) and the National Family Health Survey (NFHS) data sets, these are repeated cross-sections, and the existing literature on SRH with these data in India is relatively limited.

We obtain foundational facts about ill health for individuals in India at one point in time (calendar years 2018 and 2019). We study the variation of ill health with age, and the impact of a variety of socioeconomic factors such as income, gender, caste, religion and education.

We explore the variation of SRH by location. The dataset divides the country into 102 ‘homogeneous regions’ (HRs). In myriad other contexts, the evidence shows substantial heterogeneity within the country, across the HRs. We explore geographical heterogeneity in ill-health, and in the impact of income upon health.

Ultimately, multiple health outcome measures—medical tests, SRH, mortality—need to come together into a rich literature on the causes and consequences of ill health, which can inform the decision making of individuals, health care providers, and public health. The CPHS longitudinal data makes possible a new literature where the causes and consequences of health can be explored. This paper constitutes a first building block for that research program.

## Methods

### Ethics statement

The data used in the paper is sourced from Consumer Pyramids Household Survey database which is collected by the Centre for Monitoring the Indian Economy (CMIE). We did not seek IRB approval since the process of data collection was not undertaken by us. CMIE takes the consent of every household that participates in the household survey.

### Data sources

We use data from the Consumer Pyramids Household Survey (CPHS) carried out by the Centre for Monitoring Indian Economy (CMIE). This is available to all researchers upon payment of a subscription fee. The data can be sourced from https://consumerpyramidsdx.cmie.com. (Queries related to subscription may be sent to Mr. Prabhakar Singh, Head, Institutional Business, CMIE. sprabhakar@cmie.com.) The CPHS collects high-quality data through face-to-face interviews with households. Answers provided by respondents are captured in a mobile phone through a specially developed app. CPHS measures a panel of households at three points in each year. Households are met with in three waves a year; Wave 1 runs from January—April, Wave 2 from May—August, and Wave 3 from September—December.

The sample is nationally representative and selected through a multistage stratified design. The count of sample households increased from 166,744 households in the January—April, 2014 wave to 174,405 households in the September—December, 2019 wave. Over the six years a total of 65,892 households were added to the original sample of 166,744 households and 58,231 households were dropped for various reasons. The broadest level of stratification is the “Homogeneous Region (HR)”, a set of neighbouring districts that are similar in three dimensions: agro-climatic conditions, urbanisation levels and female literacy. India is partitioned into 102 such HRs in the CPHS. The HR is further divided into stratum—which is either rural (all villages in the HR) or urban (towns which are further classified into four different stratum based on their size) region within a HR. Towns with more than 200,000 households are classified as *Very Large* stratum, between 60,000–200,000 are classified as *Large* stratum, between 20,000–60,000 households are *medium* stratum and below 20,000 households are *small* stratum. Thus, each HR is further disaggregated into 5 stratums, 1 rural and 4 urban. The primary sampling units (PSUs) in the survey are villages and towns from the 2011 Census of India. The ultimate sampling units are the households from these primary sampling units. CPHS questions are of two broad categories: those that are asked at the household level (such as expenditure, income and asset ownership) and those that are asked of each member of the household (such as demographics, religion, caste, education, and health).

In any narrow period of time, there can be special problems like a local epidemic or a natural disaster, which can generate higher ill-health in one particular region. Some aspects of the disease burden can be seasonal; e.g. reduced water quality in summer or respiratory ailments in North India in the winter. In the year 2017, there is the possibility of an adverse impact upon health of the Demonetisation event of November 2016. On 8 November, 2016 the government of India banned currency denominations of Rs.500 and Rs.1000, wiping out 86% of the currency overnight. These notes were replaced by newer notes of denominations of Rs.500 and Rs.2000. In the year 2020, there was the pandemic. Hence, in this paper, we use data from the six waves of 2018 and 2019. Averaging over six waves helps remove isolated episodes of ill health and seasonal factors. The results in this paper can be viewed as a characterisation of baseline conditions in India, against which special periods such as the pandemic can be compared in future research.

The CMIE measurement process thus involves one member—the respondent—who responds on behalf of all members in the household. The text of the question is *“Does the member feel healthy as of today?”*. To that extent, this measure is not *self*-reported health, but the state of health of an individual, as observed by the survey respondent in the same household. This may help ensure that relatively minor conditions, which are known to the person but not the respondent, do not influence the answer. Similarly, mental health issues could influence SRH as reported by an individual but not the information obtained from an observer in the household.

### Variables of interest

**Age** We form six discrete age bins: 0–4, 5–9, 10–34, 35–49, 50–59 and 60+. We expect a U-shaped curve where the very young and the very old are more unwell. This is because infants have a weak immune system and suffer from a high incidence of infectious disease. The elderly are gradually brought down by the process of aging.
**Social and demographic characteristics** Aggregate facts about the incidence of ill health will be shaped by social as well as demographic characteristics. Towards this, we examine the variation in the age-specific SRH rate across gender, education, caste, and religion.
**Income** Income could influence health through four pathways: nutrition, housing quality (which would influence hygiene and physical access for disease vectors), knowledge and health care. In this paper, we seek to measure the overall correlation between income and ill-health, summing across these four factors. There are always concerns about adequately capturing the income of a household through a survey. For example, [31] presents a discussion on the challenges of measurement through a household survey. However, it is the best available measure of cash-flows of households, and their ability to seek better preventive health as well as health-care. In order to avoid endogeneity bias (where ill-health triggers off reduced income), income in month *t* is expressed as an average of income over the previous 12 months.
  We use the total income of the household reported in the CPHS. It is the summation of total income of every earning member and the income of the household collectively, which cannot be attributed to any individual member. This includes income received from all sources such as rent, income earned from self production, private transfers, wages, overtime, bonus, etc. Household income as reported in CPHS is converted into real rupees using the Consumer Price Index. The base year of CPI is 2012–13.
**Location** We examine how ill health varies by location. This is interesting, in and of itself, as it shows where the unwell persons are. This shows the spatial distribution of health care requirements. In addition, this can potentially yield insights on public health interventions that can improve health.

### Odds ratios of ill health

We estimate a pooled logit model explaining ill-health. Standard errors are clustered at the level of the “primary sampling unit (PSU)”. These are the towns for the urban sample and villages for the rural sample. We estimate three models:

1. We first model the log odds of reporting poor health using binary regression with a logit link function and robust error variance, given as:
  $$log\frac{\pi_{i}}{\left(1-\pi_{i}\right)}=\beta_{0}+\beta X_{i}+T$$
  where $\frac{\pi_{i}}{\left(1-\pi_{i}\right)}$ is the odds that self reported poor health for individual *i* = 1, 0 otherwise. *β*_0_ represents the log odds of reporting poor health for the reference category. *βX*_*i*_ represents the change in the log odds of reporting poor health for a one unit change in a vector of independent variables (age, sex and education, income quintiles). Here, the odds of an event is the ratio of the probability of the event happening to the probability of not happening (i.e $\frac{p}{1-p}$).
  In this model, we have time fixed effects, *T*, to permit systematic change of the overall ill-health rate by wave.
2. The location may influence ill health. This could derive from differences in state performance on public health, e.g. on problems such as pollution control. To measure the variation of ill-health by location, after controlling for individual characteristics, we allow the intercept to vary by location (*k*). We estimate:
  $$log\left(\frac{\pi_{i}}{1-\pi_{i}}\right)=\beta_{0}+\beta X_{i}+k+T$$
  This HR fixed effects estimate effectively has a distinct intercept *k* for each homogeneous region. This yields estimates for 102 coefficients for the HRs of India, which can be viewed as properties of these locations. A sorted list of these coefficients represents a sorted list of the places in India where certain features of these locations are associated with the best or worst health, after controlling for household characteristics.
3. Finally, we also allow the slope in income to vary by location. We estimate:
  $$log\left(\frac{\pi_{i}}{1-\pi_{i}}\right)=\beta_{0}+\beta X_{i}+k+k*log.income+T$$
  With this in hand, there is an overall model that applies for the whole country, but the slope and intercept for log income are measured on a per-HR basis.

With these estimates in hand, we perform certain counter-factual calculations for one canonical individual. We focus on a Hindu male, who is of the SC/ST caste, who is in the 35–49 age group, and is educated upto Class12/Diploma level. The person is in a rural household which has a real income of Rs.13,000 per month (about USD 266 per month). This is approximately the modal individual in the dataset. Holding these individual and household characteristics constant, we explore the extent to which the predicted ill health probability changes when this person is moved across each HR. This gives us a way to visualise the impact of location on SRH. It shows how ill health varies within India, after removing non-comparability owing to differences in age and income.

## Results

The data contains information on 736,945 unique individuals from 170,804 unique households across the six waves from Wave 1 2018 till Wave 3 2019. The overall sample size is 3.5 million observations: on average, each unique individual is measured 4.5 times. S1 Table in S1 File shows the sample size across each wave used in the dataset. The panel is unbalanced: some households are present in less than three waves.

S2 Table in S1 File presents summary statistics about the dataset. There are 47.18% females. About 69.5% of the sample is in the 10–49 age group. While 84% of the sample is Hindu, 11% is Muslim. The education measure that we focus on in this paper is the education level of the most educated person in the household: this person may be expected to process information and help make health-related decisions for everyone in the household.

The overall estimate of self-reported ill health rate in the dataset is 3.25%. This overall average SRH rate implies an estimated 44.4 million individuals in India were reported as unhealthy on any one given date. It also implies that, on an average, individuals are unwell for about 12 days a year.

**Fig 1:**
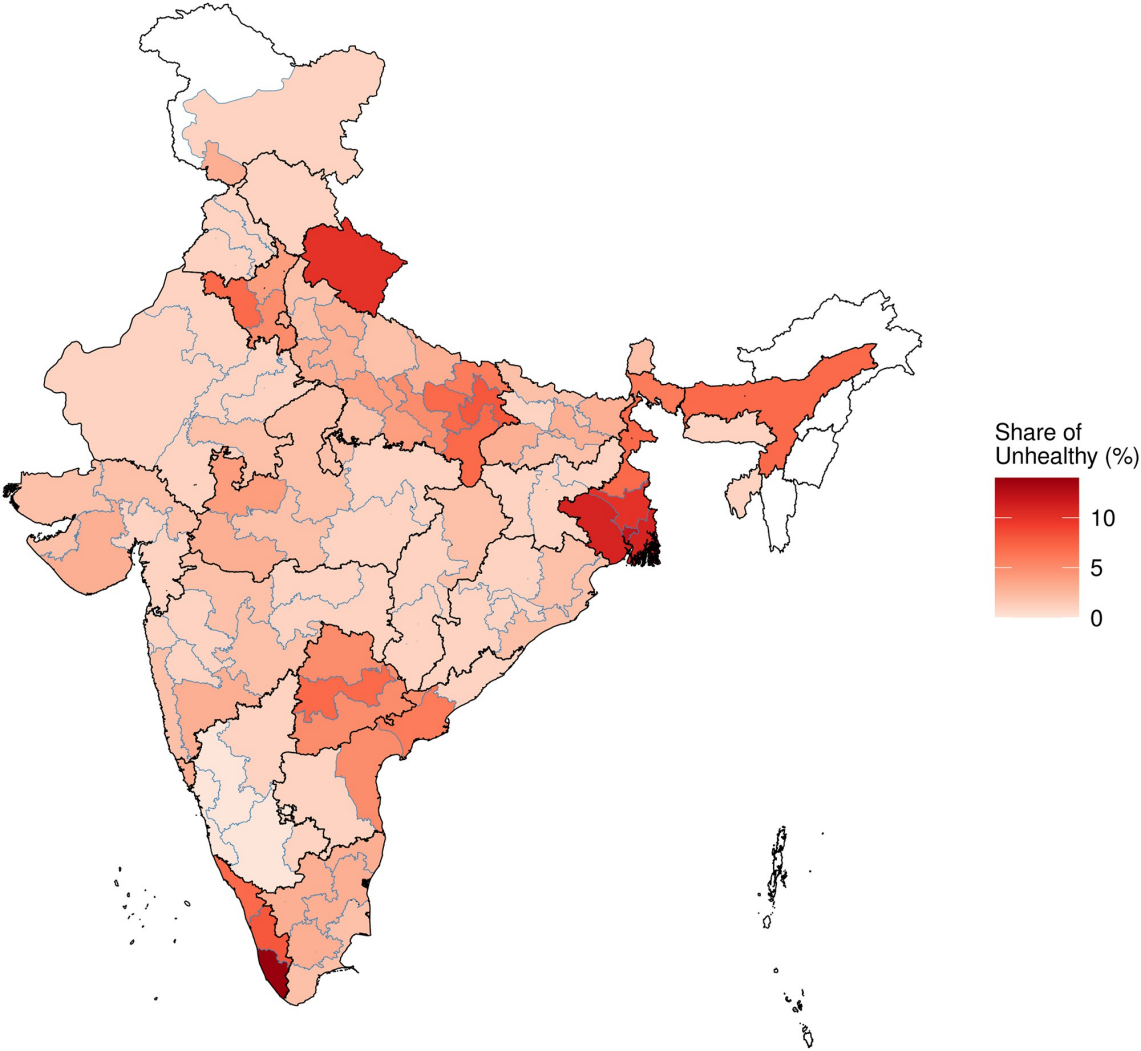
Share of unhealthy people across HRs.

Fig 1 presents the share of unhealthy people in every HR. The map shows many intriguing facts that invite greater exploration. A few regions stand out as having a greater ill-health rate: Uttarakhand, West Haryana, East Uttar Pradesh, Bengal, Assam, Telangana and Andhra Pradesh, and Kerala. Both Himachal Pradesh and Uttarakhand are mountainous regions, but ill health in Uttarakhand is much worse.

One concern about SRH measurement is the extent to which psychological biases are systematically present in certain cultures. When we look at the map, there are many states within which we may expect a certain degree of cultural homogeneity, but the SRH values are heterogeneous. Haryana, Kerala, Uttar Pradesh and West Bengal show such features. This helps increase our confidence in the extent to which the psychological aspects of SRH measurement are not primarily shaped by culture.

Fig 1 shows the rates of self-reported ill health across the country, regardless of the reason why this might be happening. This map directly illuminates health *care* requirements. From the viewpoint of understanding the health of the people, of course, we must recognise that many factors are at work in generating this heterogeneity. As an example, high levels of ill health in Kerala may reflect the greater share of the elderly in Kerala.

**Fig 2:**
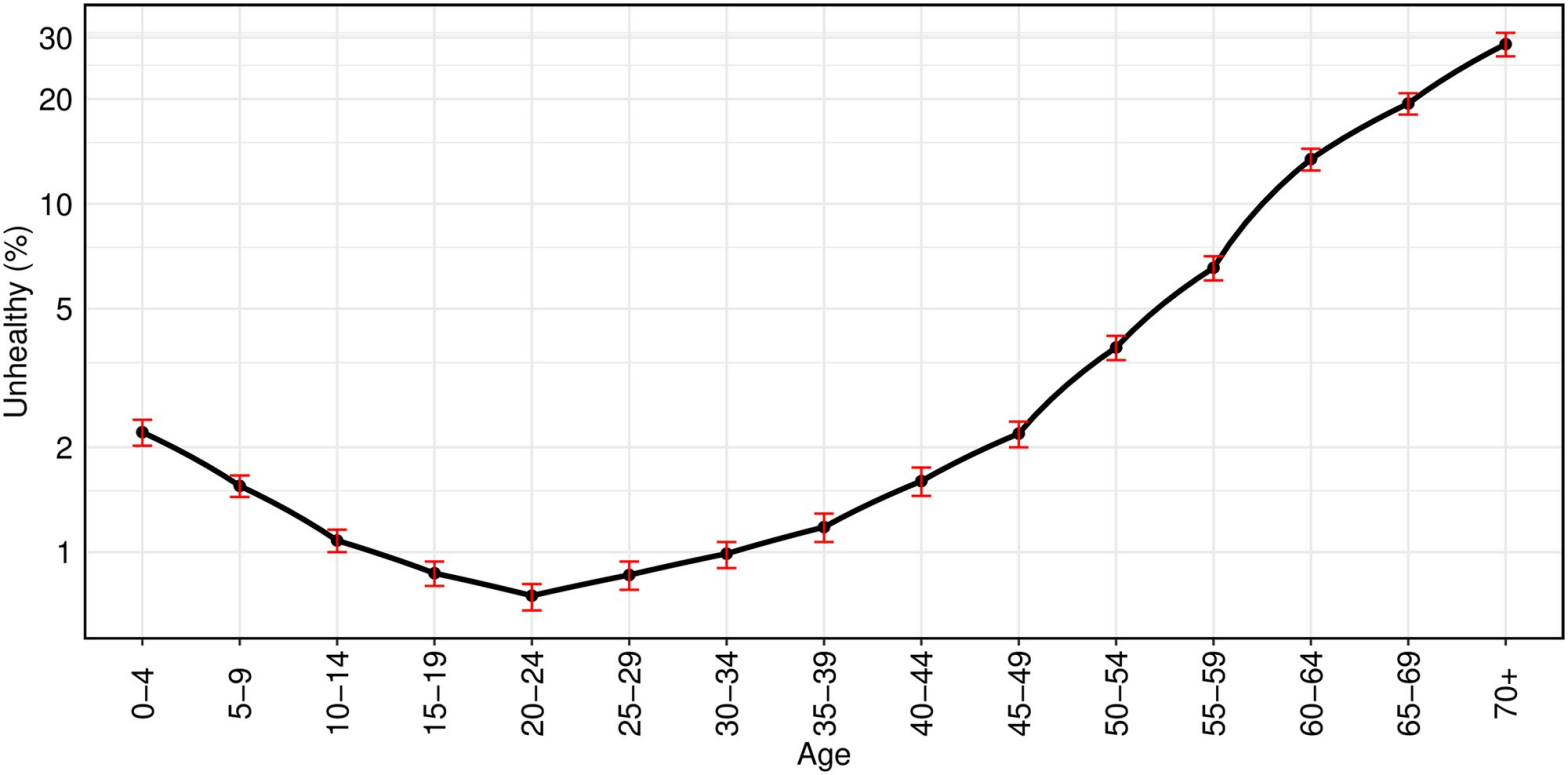
Age specific unhealthy curve.

We turn to examining the variation of the ill-health rate by individual and household characteristics. Fig 2 shows the age variation of the ill-health rate. It is useful to recall that an ill health rate of 1% corresponds to 3.65 unhealthy days per year. The graph, where the *y* axis is in log scale, shows a U shaped pattern. A little over 2% of infants (0–4 age group) are reported as unhealthy. This declines to the lowest ill-health at the 20–24 age group, and then degrades with age. For the entire age range from 10 to 39 years, the ill health rate is relatively low, with a peak value that is slightly above 1%. As we move to the elderly, there are large jumps in the share of unhealthy people.

Table 1 shows the fraction of people who reported being unhealthy in each age group, interacted with socio-economic characteristics. The first row—Overall—represents a useful comparison point for the other rows.

**Table 1:** Fraction of persons who report ill-health.

	Age					
	0–4	5–9	10–34	35–49	50–59	60+
Overall	2.21	1.55	0.91	1.66	5.1	20.24
Region						
Rural	2.26	1.59	0.98	1.82	5.32	20.67
Urban	2.04	1.44	0.74	1.36	4.70	19.52
Gender						
Male	2.22	1.53	0.86	1.43	4.60	20.59
Female	2.20	1.56	0.96	1.89	5.66	19.82
Religion						
Hindu	2.21	1.55	0.90	1.59	5.07	20.22
Muslim	2.29	1.57	1.06	2.73	6.69	22.72
Others	1.90	1.45	0.54	0.60	2.37	16.98
Caste Category						
Upper Caste	2.15	1.54	0.94	2.22	6.57	24.21
OBC & Intermediate Caste	2.22	1.60	0.88	1.41	4.43	19.00
SC & ST	2.23	1.48	0.95	1.79	5.42	18.92
Not Stated	2.61	1.53	0.58	0.57	1.98	22.02
Maximum household education						
None or Primary	2.73	1.70	1.23	2.08	6.02	21.70
Class 10	2.10	1.50	1.02	1.74	5.26	19.59
Class 12/Diploma	2.20	1.46	0.81	1.67	4.82	19.51
College & above	2.21	1.69	0.82	1.45	4.94	20.90
Income Quintiles						
Lowest	2.21	1.60	1.23	2.49	6.87	21.82
Second	2.12	1.47	0.94	1.61	4.91	19.46
Middle	2.26	1.33	0.81	1.34	4.92	20.88
Fourth	2.23	1.66	0.69	1.24	4.52	20.42
Fifth	2.31	1.78	0.63	1.00	3.85	17.65
Region						
Central	2.46	1.47	0.56	0.73	2.77	13.93
East	2.15	1.61	1.16	3.34	8.89	23.73
North	2.26	1.53	0.93	1.71	5.32	24.06
North-East	2.02	0.83	0.95	4.13	12.95	39.05
South	1.61	1.41	0.59	0.51	3.68	24.74
West	2.69	1.87	1.02	1.04	1.61	4.27
Sample Size	83,135	192,377	1,569,556	852,713	450,194	333,305

The table presents the ill health rate in different age groups, with the variation across socio-economic characteristics, in India.

In the 35–49 age range, there is greater ill-health with the rural population, for women and for Muslims. The upper caste shows the highest ill-health rate in the 35–49 age range. When the most educated person in the household is in the lowest education category (none or primary), the ill-health rate is much higher, at 2.08%, in the 35–49 age range and 1.23% in the 10–34 age range.

Higher household income is generally associated with lower ill health rates. For all the age ranges from 10 to 59, there is a monotonic relationship: richer households have reduced ill health. But this is not the case below age 10 and above age 59.

A survivorship bias may be at work. If poor people are more prone to die, when prosperity arrives, mortality may improve, so that persons are more likely to be alive and report they are unwell. The ill-health rate for age 0–4 is at 2.21% in the lowest income quintile and actually worsens to 2.31% at the top quintile. Similarly, when it comes to the elderly, the ill health rate falls to 19.46% in the second income quintile, but rises to 20.88% for the middle and then the best value of 17.65% is obtained at the top income quintile.

Finally, there are strong location effects visible in this table. In the age 35–49 range, we get a large variation by region. Two regions are well above the overall average of 1.66% (4.13% (North-East), 3.34% (East)) and three are well below (1.04% (West), 0.73% (Central) and 0.51% (South)).

While the patterns in Table 1 are quite revealing, all these covariates are correlated with each other. It is, therefore, useful to look at the adjusted odds ratios from the logit regression where these covariates are all present at once in the model. These are presented in Table 2. Columns (1) presents the odds-ratio and the 95% confidence intervals and clustered standard errors for a single model that covers all households, with only wave (time) fixed effects. Column (2) presents the same, with HR fixed effects. The single intercept for the whole country is broken out into 102 intercepts for the 102 HRs. Finally, Column (3) presents the results with HR controls and HR and income interaction effects. Instead of a single coefficient for log income for all households, this coefficient is permitted to vary by HR.

**Table 2:** Odds ratios from logistic regressions that predict self reported (ill)Health.

	(1)	(2)	(3)
Residence *(Ref:Rural)*			
Urban	1.064	1.039	1.025
	(0.936, 1.210)	(0.975, 1.108)	(0.961, 1.093)
Age *(Ref: age 10–34)*			
0–4	2.771***	2.595***	2.587***
	(2.525, 3.042)	(2.346, 2.871)	(2.337, 2.863)
5–9	1.814***	1.814***	1.826***
	(1.704, 1.932)	(1.695, 1.943)	(1.706, 1.955)
35–49	1.754***	1.780***	1.788***
	(1.612, 1.909)	(1.621, 1.955)	(1.629, 1.963)
50–59	5.859***	6.065***	6.043***
	(5.303, 6.472)	(5.457, 6.740)	(5.438, 6.715)
60+	28.071***	31.974***	31.639***
	(25.269, 31.185)	(28.710, 35.610)	(28.423, 35.219)
Gender *(Ref: Male)*			
Female	1.053***	1.051***	1.048***
	(1.022, 1.086)	(1.019, 1.084)	(1.016, 1.081)
Religion *(Ref: Hindu)*			
Muslim	1.062	1.058	1.051
	(0.953, 1.183)	(0.989, 1.131)	(0.984, 1.122)
Other	0.725***	1.038	1.029
	(0.595, 0.884)	(0.952, 1.131)	(0.943, 1.123)
Caste *(Ref: Upper Caste)*			
Not Stated	0.747	1.031	1.049
	(0.531, 1.050)	(0.915, 1.162)	(0.927, 1.186)
OBC/Intermediate	0.705***	0.989	0.995
	(0.641, 0.776)	(0.944, 1.036)	(0.951, 1.040)
SC/ST	0.730***	0.974	0.983
	(0.666, 0.800)	(0.927, 1.023)	(0.936, 1.032)
Education *(Ref: None or Primary)*			
Class 10	0.976	1.016	1.070***
	(0.906, 1.050)	(0.966, 1.070)	(1.017, 1.125)
Class 12/ Diploma	0.964	0.981	1.037
	(0.879, 1.056)	(0.924, 1.041)	(0.977, 1.101)
College and above	1.070	1.014	1.063
	(0.955, 1.200)	(0.945, 1.089)	(0.991, 1.140)
Log.income	0.788***	0.839***	1.162
	(0.730, 0.851)	(0.798, 0.883)	(1.006, 1.342)
Intercept	0.094***	0.008***	0.0004***
	(0.047, 0.186)	(0.005, 0.014)	(0.0001, 0.002)
HR controls	No	Yes	Yes
HR*log income controls	No	No	Yes
Wave (Time) controls	Yes	Yes	Yes
Observations	3,481,280	3,481,280	3,481,280
Log Likelihood	−402,203.200	−364,715.000	−363,194.600
Psuedo R2	0.189	0.265	0.268
BIC	804737.8	731282.7	729763.2

*Note*:

*p<0.1;

**p<0.05;

***p<0.01

The table presents the results from a logit regression of individual characteristics on self reported health. We report the odds-ratio and the 95% confidence intervals. Standard errors have been clustered at the PSU level.

The variation of the odds-ratio in Model (2), by age, is consistent with the variation seen in the simple summary statistics of Fig 2. The coefficient of log income is statistically and economically significant. A striking feature of Model (2) when compared with Model (1) is that once we control for age, income and location, the importance of religion and caste subsides. The most important sources of variation of SRH, in Model (2) and (3) are age, income and location. In Model (3), there are small enhanced ill health rates for women (OR = 1.048) and one education categories (OR = 1.070 for Class 10).

When we compare Model (1) versus Model (2) there is a large gain in the Bayesian Information Criterion (from 804,737 to 731,282). This suggests that the HR fixed effect (variation of the intercept of the model by HR) is particularly important. In comparison, there is a reduced gain in going from Model (2) to Model (3) (from 731,282 to 729,763).

**Fig 3:**
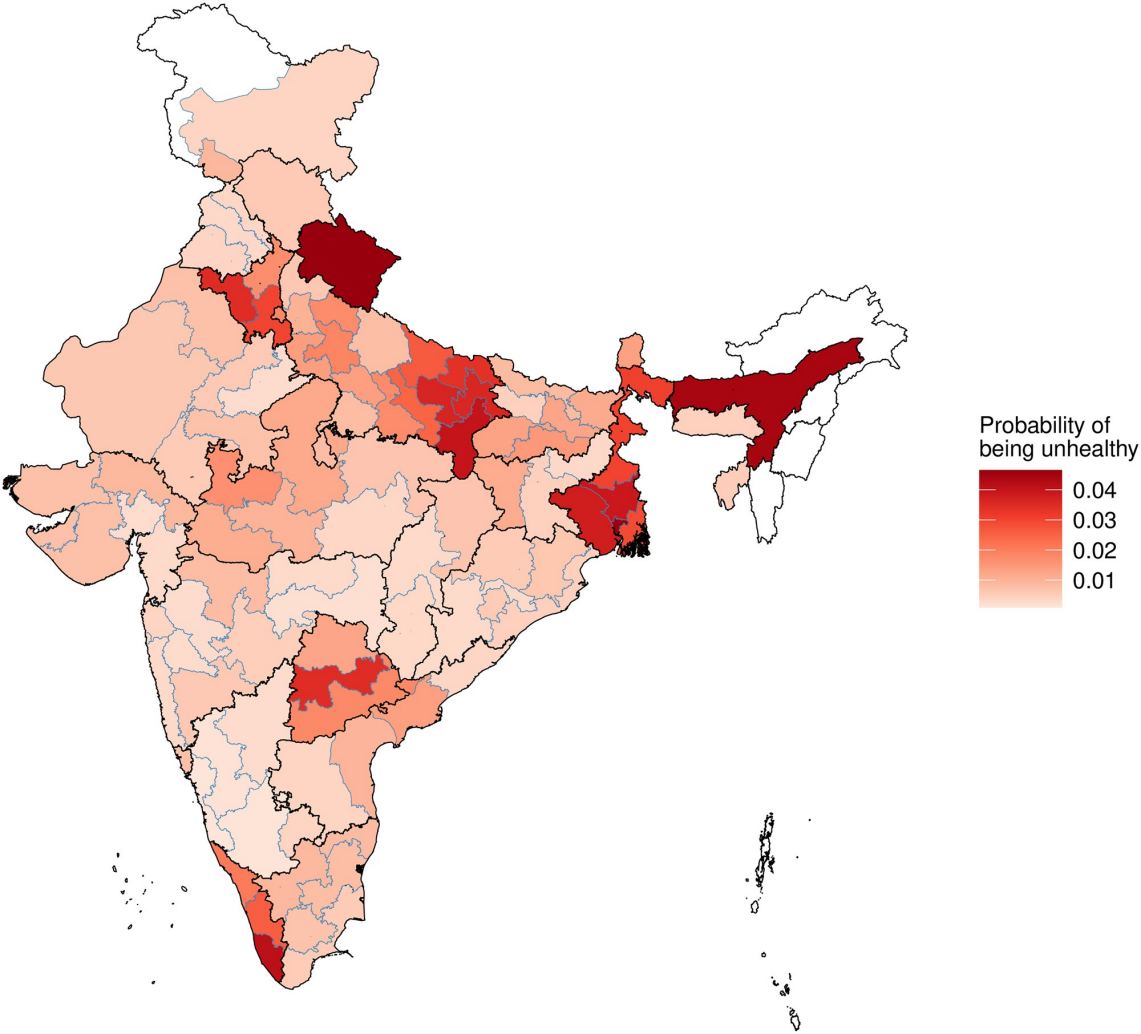
Model-based predictions of the probability of ill health for a fixed individual.

Fig 1 had shown us the unconditional geographical variation of ill health. With the models of Table 2 in hand, we can control for some explanatory variables and focus on geographical variation. In order to do this, we make predictions for the ill-health rate for the canonical individual: a Hindu, male, in the 35–49 age group, of the SC/ST caste category, with a Class 12/Diploma education, in a rural household with a real income of Rs.13,000 per month. This is approximately the modal person in the dataset. Within each homogeneous region, we compute the predicted probability of ill health, using Model (3). These predicted probabilities are mapped in Fig 3.

The comparison between the unconditional geographical variation of ill health (Fig 1) and the geographical variation of ill-health after controlling for individual characteristics (Fig 3) is revealing. The range of values, in the unconditional map, is much higher, with values ranging from 0 to 15%. Once we narrow down to the canonical person, important sources of variation (age and income) are removed from the picture. Persons in the 35–49 age range are healthier than the overall population. Hence, the range of values seen in Fig 3 is smaller: from 0 to 4%.

When a hot spot like south Kerala is visible in Fig 1, this could be associated with age structure, income or location. When it shows up (more weakly) in Fig 3, this suggests there is some feature of the *location* which is associated with greater ill health, which is not merely induced by age structure and income.

A striking feature of the two figures is the extent to which they look similar (though of course the scale is quite different). This is consistent with the statistical findings in Table 2, that location has a powerful impact upon ill health.

**Fig 4:**
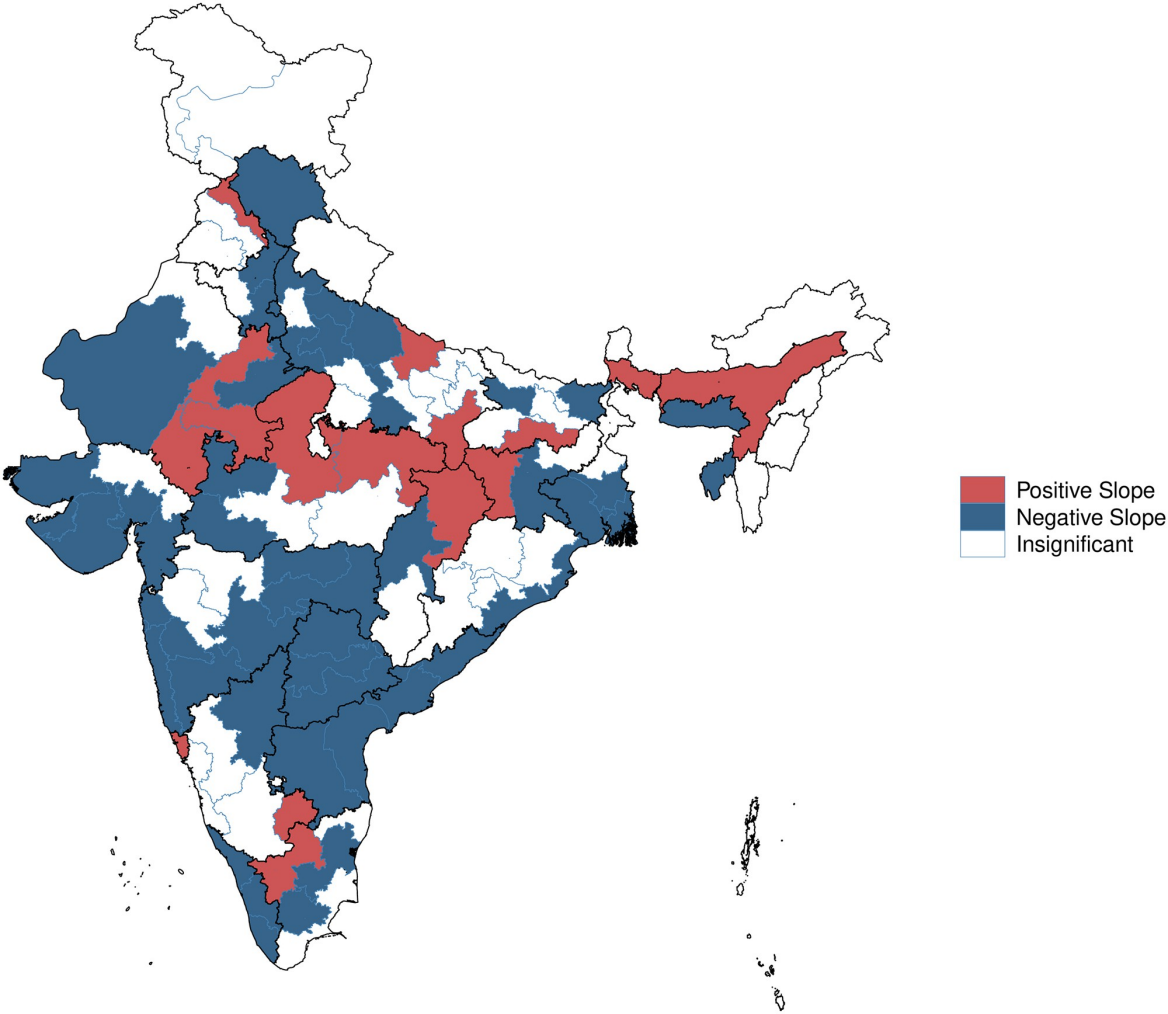
Variation in income and HR slope coefficients.

The overall result in Model 2 and Model 3 is that health improves with log income. Model (3) permits the slope in income to also vary by location, thus yielding 102 coefficients, for the slope on log income in each HR. Fig 4 helps us visualise the estimated slopes. Regions that are coloured blue are those where higher income is associated with reduced ill health, where the null hypothesis of a slope of 0 is rejected at a 95% level of significance. This property is found in only 49% of the HRs of India. With 35% of the HRs, *H*_0_ is not rejected. In 16% of the locations, shown in red, ill health is *greater* for households with higher income. We also test for the non-linearity between income and ill-health through a cubic spline based regression. The predicted probability of ill health is seen to linearly decline with income as presented in S1 Fig in S1 File.

## Discussion

The paper finds that there is a U-shaped relationship between age and self-reported ill health. This is similar to previous research that finds that a sizable number of elderly who report a poor self-rated health [32]. Women have worse self-related health status, also consistent with other literature [33]. The conventional wisdom of health economics expects that higher income generates better health, through a combination of improved nutrition, housing (which impacts on hygiene and disease vectors), knowledge and health care [34]. In the aggregate, in our results, an increase in log income is indeed associated with reduced ill health. While this paper makes no causal claims, ultimately, if there is a causal and positive link between income and health, then mere economic growth would help improve health (while recognising that some of this impact flows through higher public and private expenses on health).

However, the interesting finding of the paper is the role of location in determining health. In fact, after location, several of the social variables such as caste and religion lose their statistical significance. We find that there is a large variation in the self-reported health of the identical person depending on the location of the person. Table 3 shows the names of the 10 HRs of India with the highest and lowest ill health rates. We may offer some conjectures about the epidemiological phenomena at work in Fig 3. The region of eastern Uttar Pradesh, Assam and Bengal is known to face high arsenic contamination of water [35, 36]. The adverse health outcomes of arsenic contamination in the form of skin lesions, neurological effects, obstetric problems, cardiovascular effects and cancers typically involving the skin, lung, and bladder [37–39]. Our reported measures of poor health in Eastern India may reflect this phenomenon.

**Table 3:** The HRs with the highest and lowest ill health rates.

Rank	By raw ill-health rate	Predicted modal person
1.	Chitradurga—Mysore	Uttara Kannada—Dakshin Kannada
2.	Uttara Kannada—Dakshin Kannada	Chitradurga—Mysore
3.	Belgaum—Shimoga	Belgaum—Shimoga
4.	Bharatpur—Tonk	Hingoli—Gadchiroli
5.	Hingoli—Gadchiroli	Ahmadabad—Kheda
6.	Giridih—Dumka	Nashik—Ahmadnagar
7.	Bidar—Bellary	Bidar—Bellary
8.	Ahmadabad—Kheda	Bharatpur—Tonk
9.	Pune	Pune
10.	Rajasamand—Banswara	Amravati
93.	Assam	Azamgarh—Gorakhpur
94.	Sirsa—Bhiwani	Faizabad—Jaunpur
95.	Azamgarh—Gorakhpur	Sirsa—Bhiwani
96.	Palakkad—Idukki Azamgarh—Gorakhpur	Puruliya—Medinipur
97.	Uttarakhand	Mau—Sonbhadra
98.	Barddhaman—Nadia	Barddhaman—Nadia
99.	Puruliya—Medinipur	Kottayam—Thiruvananthapuram
100.	24 Parganas	Assam
101.	Kolkata—Haora	Kolkata—Haora
102.	Kottayam—Thiruvananthapuram	Uttarakhand

The first column shows the 10 HRs of India with the lowest and highest observed ill-health rates (i.e. from the picture seen in Fig 1). This reflects a combination of income, age structure and location characteristics.

The second column shows the 10 HRs of India with the lowest and highest predicted ill-health rates for the modal person (i.e. predicted using Model (3) of Table 2, which is the same as the geographical variation depicted in Fig 3). This reflects location characteristics.

As an example, Uttarakhand shows up as the 98th most unhealthy HR based on the raw ill-health rate. After controlling for income and age structure, it proves to have the highest ill-health.

Similarly, the high ill health in Uttarakhand, as opposed to the neighbouring hilly state of Himachal Pradesh, might be associated with religious pilgrimage and festivals [40, 41] where pilgrims from all across India bring novel pathogens to Uttarakhand, and unhygienic crowding of pilgrims within Uttarakhand create conditions where more virulent strains succeed. There is a need for further research in understanding the sources of this variation, particularly the variation seen in the predicted ill health rates for the modal person which reflect characteristics of these *locations* that can potentially be influenced by modified strategies in public health.

The second surprising result is that there is considerable geographical heterogeneity in this slope. In about 40% of India, the relationship is negative (the rich have less ill health). But in the rest of India, this association is not observed. There is a significant area where health is *worse* for higher income households. The two maps (Figs 3 and 4) also do not fit within a simple North India vs. South India stereotype. While the population centres of Uttar Pradesh and Bihar are considered to be poverty traps, the canonical person is only particularly unhealthy in Eastern Uttar Pradesh. There are some parts of UP and Bihar where the rich are healthier than the poor. The HRs which have greater difficulties are present in many parts of the country and call for a revision of our priors about where ill health in India is found. The phenomena seen in the two maps (Figs 3 and 4) are not the same. The regions where ill health worsens for high income people are not the same as the regions with high ill health. These are distinct phenomena that require distinct explanations.

The impact of increased income upon health through improved nutrition and housing is likely to operate all across the country in relatively uniform ways. If health care is completely absent, there should be a negative slope in income as higher income is likely to generate better nutrition, housing and knowledge. If government-supplied health care works well, all income groups would get access to comparable health care, and a negative slope in income would still be generated through the impact of nutrition, housing and knowledge. If private health care works well, higher income would generate better purchases of health care services, and a negative slope in income would arise. It is thus a puzzle, to explain the white areas (rich and poor are similarly healthy) and red areas (the rich have higher ill-health).

We conjecture that this is related to health care and survivorship bias. High income households in certain regions may get low quality care, to a point where it overshadows the other channels of impact (improved nutrition, housing, knowledge). If there is a large income gap in mortality, for the young and the old, then high income may generate more survivors who are in a state of ill health, thus generating a zero or negative slope for health in income. Another explanation can be found in the work by [42] who finds that health improvements in poor countries are not driven by income, or knowledge or technology, but by social factors that improve health delivery, and political factors that make population health a priority.

There are two limitations of this work. The standard difficulties of SRH measurement are present here: where health is observed through an intermediating psychological filter which introduces a certain degree of imprecision. To the extent that these psychological characteristics are correlated with the variables of interest, the statistical estimates are biased. The second limitation of this study is that the logistic regressions shown here lack a causal interpretation. These calculations should be viewed as merely describing features in the data. They do not guide interventions where certain features are changed in order to impact upon SRH.

## Conclusions

In this paper, we have undertaken a novel examination of a health outcome measure in India in a large-scale household survey database pertaining to calendar years 2018 and 2019, drawing on the health status seen in 3.2 million records for individuals. In the existing health literature, there is a substantial analysis of the health of infants, children and mothers. In this paper, we analyse the entire population.

The overall aggregate ill-health rate is about 3.25%, which maps to about 44 million persons being unwell on any one day. The average individual is unwell for about 12 days a year. There is a U-shaped curve, with a long zone of reduced ill health rates from age 10 to age 39.

The important correlates of SRH are age, income and location. Once these are controlled for, other individual characteristics that were explored here (education, caste, religion, gender) are relatively unimportant.

Location is remarkably important. Health researchers, and health policy makers, need to look beyond all-India averages, and recognise the immense variation within the country. The three maps constructed in the paper—the raw ill-health rate, the ill-health rate of the modal person and the regions where higher income is correlated with improved health—are novel and go beyond the simple preconceptions of North India vs. South India. The geographical variation shown here merits further exploration.

## Supporting information

S1 File (PDF)
