# Supplementary material for: Distribution of self-reported health in India: The role of income and geography
Source: PLoS One. 2023 Jan 27;18(1):e0279999. doi: 10.1371/journal.pone.0279999 (PMC9882784; doi:10.1371/journal.pone.0279999)
Supplement: S1 File — (PDF) [file pone.0279999.s001.pdf]

1 Supporting information

Table S1: Sample size across the three waves of 2018 and 2019

Months	Wave	Total Households	Total Members
Jan - Apr, 2018	W1 2018	143151	575082
May - Aug, 2018	W2 2018	149101	594655
Sep - Dec, 2018	W3 2018	147123	584109
Jan - Apr, 2019	W1 2019	146292	582180
May - Aug, 2019	W2 2019	147840	575044
Sep - Dec, 2019	W3 2019	147291	570210

This table presents summary statistics about the CPHS data for 2018 and 2019.

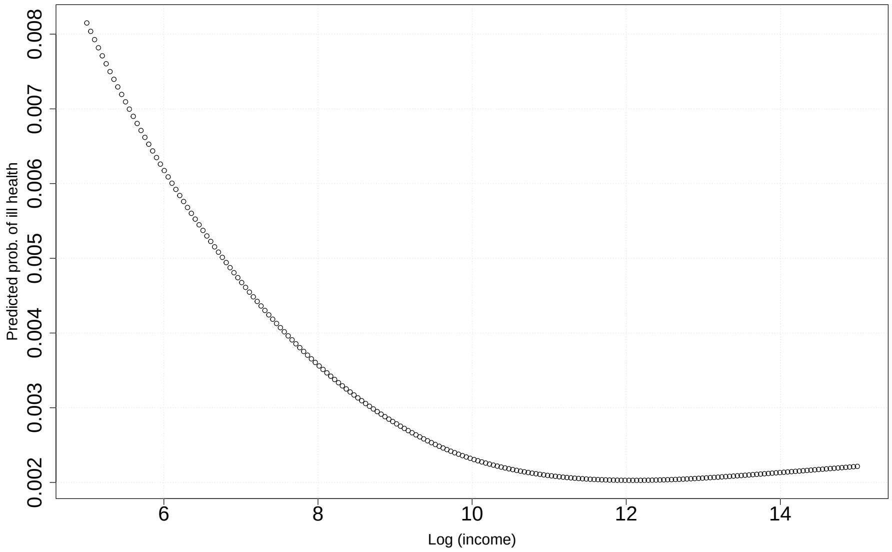

Fig S1: Predicted probability of ill health from a cubic-spline model

Table S2: Data Summary: 2018 and 2019

Variable		Sample Size
Unique Households		170,804
Unique Individuals		736,945
Total Sample size		3,481,280
Self Reported Health (%)		
Healthy	96.77	3,368,947
Unhealthy	3.23	112,333
Gender (%)		
Male	52.82	1,838,657
Female	47.18	1,642,623
Residence (%)		
Rural	36.86	1,283,068
Urban	63.14	2,198,212
Age Group (%)		
0-4	2.39	83,135
5-9	5.53	192,377
10-34	45.09	1,569,556
35-49	24.49	852,713
50-59	12.93	450,194
60+	9.57	333,305
Religion (%)		
Hindu	83.83	2,918,231
Muslims	10.55	367,321
Others	5.62	195,728
Caste Category (%)		
Upper Caste	23.51	818,278
OBC/Intermediate	48.36	1,683,578
SC/ST	26.66	928,222
Not Stated	1.47	51,202
Max HH Education (%)		
None or Primary	4.54	158,205
Class 10	32.21	1,121,262
Class 12/ Diploma	31.36	1,091,790
College and above	31.89	1,110,023
Income quintile (average household monthly income)		
Lowest	6273	695,718
Second	9465	696,396
Middle	12889	696,395
Fourth	18674	696,397
Highest	38968	696,374
Region (%)		
Central	8.02	279,171
East	18.53	645,021
North	34.56	1,203,178
North-East	2.39	83,115
South	19	661,390
West	17.51	609,405

This table presents summary statistics about the CPHS data for 2018 and 2019.

Acknowledgements

We thank Subhamoy Chakraborty, Moumita Das and Mithila Sarah for research assistance. We are grateful to Indu Bhushan and Amrita Agarwal for useful discussions. All errors are our own.
